# Cardiac echocardiographic analysis with multi-scale effective fusion module: a novel stroke prediction approach

**DOI:** 10.1186/s12880-026-02460-7

**Published:** 2026-05-28

**Authors:** Jiachun Xie, Dianhuan Tan, Tingting Zheng, Yun Chen, Liya Wei

**Affiliations:** Shenzhen Key Laboratory for Drug Addiction and Medication Safety, Department of Ultrasound, Institute of Ultrasonic Medicine, Peking University Shenzhen Hospital, Shenzhen Peking University-The Hong Kong University of Science and Technology Medical Center, Shenzhen, Guangdong 518036 P. R. China

**Keywords:** Stroke prediction, Echocardiographic images, Feature fusion, Multi-scale effective fusion, Attention mechanisms

## Abstract

**Background:**

Stroke remains a leading cause of death and disability, and early risk stratification is critical for prevention. Existing models based only on clinical factors may miss subtle cardiac structural and hemodynamic information visible on echocardiography. We aimed to develop a multimodal stroke prediction model integrating multi-view echocardiographic images and clinical indicators.

**Methods:**

In this retrospective study, 712 hypertensive patients (10,992 echocardiographic images; 27 clinical variables) were included. Long-axis, short-axis, and apical four-chamber views were analyzed. We developed a Multi-Scale Effective Fusion (MSEF) module combining Global Feature Fusion, Multi-Feature Reconstruction, Channel Attention, and Positional Attention to improve multi-scale feature representation. Imaging features were integrated with clinical variables to build multimodal models. Model performance was evaluated on validation and test sets using accuracy, precision, recall, and F1 score.

**Results:**

The MSEF-based imaging model outperformed comparator fusion variants and achieved an accuracy of 76.8% and an F1 score of 64.7% on the test set. After integrating clinical indicators, performance further improved, with a test accuracy of 80.2% and an F1 score of 72.1%.

**Conclusions:**

The proposed MSEF-based multimodal framework improves stroke risk prediction by effectively combining echocardiographic and clinical information, and may support earlier risk identification and clinical decision-making.

**Supplementary Information:**

The online version contains supplementary material available at 10.1186/s12880-026-02460-7.

## Background

Stroke remains one of the leading causes of morbidity and mortality worldwide and a significant public health concern, with its prevalence increasing annually. It ranks among the top global health threats, as recognized by the World Health Organization [1]. The true danger of hypertension lies not in the condition itself but in its complications, which result in high rates of disability and mortality. Among these, cerebrovascular damage is particularly severe, with stroke being the most devastating consequence. Stroke is the second leading cause of death globally, and studies have shown that approximately 62% of stroke-related deaths are directly attributed to hypertension. A large-scale epidemiological study conducted in 2022 further highlighted this risk, reporting a cumulative stroke incidence of 78.9% among hypertensive patients [2, 3]. Given the profound impact of stroke on individuals and healthcare systems, its prevention and early intervention are paramount. Developing accurate and reliable methods to predict and assess stroke risk in hypertensive patients is essential for mitigating its devastating consequences.

Stroke remains one of the leading causes of morbidity and mortality worldwide, necessitating the development of accurate and reliable methods for early risk prediction and prevention. Traditional risk assessment models often rely on clinical features such as age, hypertension, diabetes, and previous cardiovascular events. While these models provide valuable insights, they may not fully capture the complexities of individual patient risk profiles [4, 5].

Previous predictive models based on clinical features have played a significant role in advancing stroke risk assessment, providing valuable tools for identifying individuals at high risk and guiding preventive strategies [6–8]. These models have effectively utilized factors such as age, blood pressure, history of atrial fibrillation, and diabetes to stratify stroke risk in various populations, contributing to better clinical decision-making [9–11]. However, despite their usefulness, these models have limitations, particularly in their ability to capture the intricate structural and functional details of the heart. They often fail to incorporate the rich information available from echocardiographic imaging, such as subtle changes in cardiac morphology, valve abnormalities, and wall motion dynamics, which can be crucial indicators of stroke risk. Therefore, the predictive capabilities of models, especially those relying solely on clinical parameters, can be significantly constrained when imaging features are instrumental in understanding stroke pathogenesis. This necessitates the integration of echocardiographic data into stroke risk prediction frameworks to facilitate a more holistic and accurate evaluation. Challenges in feature selection and representation, though evident here, are common across various domains [12–15].

Echocardiography, a widely used non-invasive imaging technique, provides comprehensive and detailed visualization of cardiac structures and functions, enabling clinicians to assess heart anatomy, chamber sizes, wall thickness, valve functionality, and blood flow dynamics [16, 17]. It plays a crucial role in diagnosing and monitoring various cardiovascular conditions, offering real-time insights into cardiac performance and assisting in the evaluation of heart failure, valve diseases, congenital heart abnormalities, and stroke risk [18]. By accurately assessing factors such as left atrial size and function, left ventricular ejection fraction, valve abnormalities, intracardiac thrombus formation, aortic atherosclerotic plaques, and potential patent foramen ovale (PFO), echocardiography offers valuable information for predicting stroke risk. Its ability to deliver dynamic and high-resolution images makes echocardiography an indispensable tool in both routine clinical practice and advanced cardiac research, aiding in the prevention and management of stroke and other cardiovascular conditions [19]. One reason for the underutilization is the complexity and variability of echocardiographic data, which often require expert interpretation to extract meaningful insights. However, advancements in machine learning and deep learning techniques have opened up opportunities to harness the predictive power of these images more effectively. By integrating echocardiographic data from long-axis, short-axis, and four-chamber views with hypertension-related clinical features, such as patient history and risk factors, it is possible to develop more accurate models for stroke risk prediction. These models can analyze subtle changes in cardiac structure and function that may not be immediately apparent to the human eye, offering a more comprehensive assessment of stroke risk. This integration has the potential to improve early detection, guide preventive interventions, and enhance personalized treatment strategies, ultimately reducing the burden of stroke in at-risk populations [20–23].

Building on this success, deep learning models have the potential to revolutionize stroke risk prediction by analyzing echocardiographic images in ways that go beyond traditional clinical assessment [24]. These models can identify intricate patterns related to cardiac morphology, wall motion abnormalities, and hemodynamic changes that are indicative of stroke risk but might be overlooked in routine examinations [25]. By combining echocardiographic data with other clinical parameters, deep learning approaches can create a more comprehensive risk stratification model, allowing for earlier and more accurate identification of individuals at high risk for stroke [26, 27]. This integration of deep learning into echocardiography could not only improve diagnostic accuracy but also facilitate personalized treatment plans, leading to better preventive strategies and outcomes for patients with cardiovascular disease. As the field continues to evolve, the application of deep learning in echocardiographic image analysis holds promise for advancing stroke prevention and enhancing our understanding of the complex relationship between cardiac function and cerebrovascular events.

Integrating echocardiographic images, including long-axis, short-axis, and four-chamber views, poses unique challenges due to the heterogeneous nature and varying scales of the data. Each view captures distinct yet complementary cardiac information: long-axis views provide insights into left ventricular function and wall motion, short-axis views offer cross-sectional perspectives on chamber morphology and structural abnormalities, while four-chamber views visualize atria and ventricles, aiding in the detection of valve dysfunction, atrial enlargement, and thrombus formation. However, traditional feature fusion techniques, such as Feature Pyramid Networks (FPN), often fail to effectively integrate these multi-view features, particularly when addressing subtle structural abnormalities or small regions of interest critical to stroke risk prediction. To overcome these limitations, we introduce advanced fusion mechanisms, including the Enhanced Feature Correlation (EFC) and proposed Multi-Scale Feature Correlation (MSFC) modules, which significantly enhance the representation and integration of multi-scale features. The EFC [28] module focuses on spatial and channel-wise correlations through its GFF submodule, which generates spatial weights to emphasize key regions and enhance feature interactions, while the MFR submodule separates and reconstructs strong and weak features, preserving fine-grained details without interference. Meanwhile, the proposed MSFC module improves the correlation between deep semantic features and shallow high-resolution features, enabling better multi-scale feature representation. By combining these modules, the proposed fusion strategy effectively captures subtle cardiac abnormalities and improves the overall predictive performance for stroke risk, particularly in cases where imaging features play a pivotal role in stroke pathogenesis.

In this study, we propose a novel approach to stroke risk prediction by integrating deep learning-based analysis of echocardiographic images with traditional clinical risk factors. Our method consists of three main components: (1) developing a deep learning model to analyze multi-view echocardiographic images (long-axis, short-axis, and four-chamber views) to enhance feature extraction and representation; (2) introducing an advanced feature fusion strategy to integrate multi-scale echocardiographic features, improving the model’s ability to capture subtle cardiac abnormalities related to stroke risk; and (3) combining the fused imaging features with hypertension-related clinical indicators to construct a hybrid predictive model, providing a comprehensive assessment of stroke risk.

We hypothesize that the integration of echocardiographic imaging data and clinical features will enhance the accuracy and robustness of stroke risk prediction. By leveraging the strengths of both deep learning and traditional clinical assessment, our approach aims to provide a comprehensive tool for early detection and intervention, ultimately improving patient outcomes.

To address the challenges associated with integrating multi-view echocardiographic data, including long-axis, short-axis, and four-chamber views, we improved traditional feature fusion strategies through the introduction of the MSFC module. While previous studies have applied the EFC module for multi-scale feature extraction, its limitations in fully capturing the spatial and semantic correlations across echocardiographic views prompted the need for further refinement [28]. The MSFC module improves upon EFC by enhancing the correlation between deep semantic features and shallow high-resolution features, allowing for better representation of multi-scale information. Specifically, the MSFC module refines spatial attention to emphasize subtle cardiac abnormalities, such as valve dysfunction, atrial enlargement, and wall motion irregularities, which are critical indicators of stroke risk. It further optimizes the integration of strong and weak features to preserve fine-grained details while mitigating feature interference, thereby improving the module’s robustness in detecting small or subtle targets within echocardiographic images.

The following sections describe the methodology for developing and validating the deep learning and clinical models, the process of integrating these models into a hybrid framework, and the evaluation of their combined predictive performance. By incorporating the MSFC module and effectively integrating imaging and clinical data, our approach provides a more accurate and comprehensive assessment of stroke risk, paving the way for enhanced prevention and management strategies.

## Methods

This study was approved by the institutional review board, and the requirement for informed patient consent was waived due to its retrospective cohort design.

### Data sources and preprocessing

This retrospective study utilized echocardiographic examination data and relevant clinical information of confirmed hypertensive patients extracted from the electronic medical record database of Peking University Shenzhen Hospital, covering the period from September 2022 to December 2023. The study included patients with comprehensive clinical and imaging data, with stroke diagnoses confirmed through neuroimaging. A total of 712 patient records were analyzed, comprising 10,992 echocardiographic images from the left ventricular long-axis, short-axis, and apical four-chamber views, as well as 27 associated clinical features. Patients were classified into positive or negative outcome groups based on whether they experienced a stroke during the course of hypertension.

The inclusion criteria were: (1) Age over 18 years; (2) Completion of echocardiographic examination with comprehensive imaging data, including long-axis, short-axis, and apical four-chamber views; (3) A confirmed diagnosis of hypertension documented in the medical history, current medical history, or disease course records; (4) Availability of complete clinical data (6) Patients admitted to the hospital between September 2022 and December 2023.

The exclusion criteria were: (1) Incomplete clinical information or missing echocardiographic images; (2) Poor-quality echocardiographic images that fail to meet diagnostic standards (e.g., unclear visualization of cardiac structures); (3) Patients with severe comorbidities that could confound stroke risk assessment, such as late-stage cancer, end-stage renal disease, or severe heart failure; (4) Patients with no documented neuroimaging confirmation of stroke diagnosis.

**Fig. 1:**
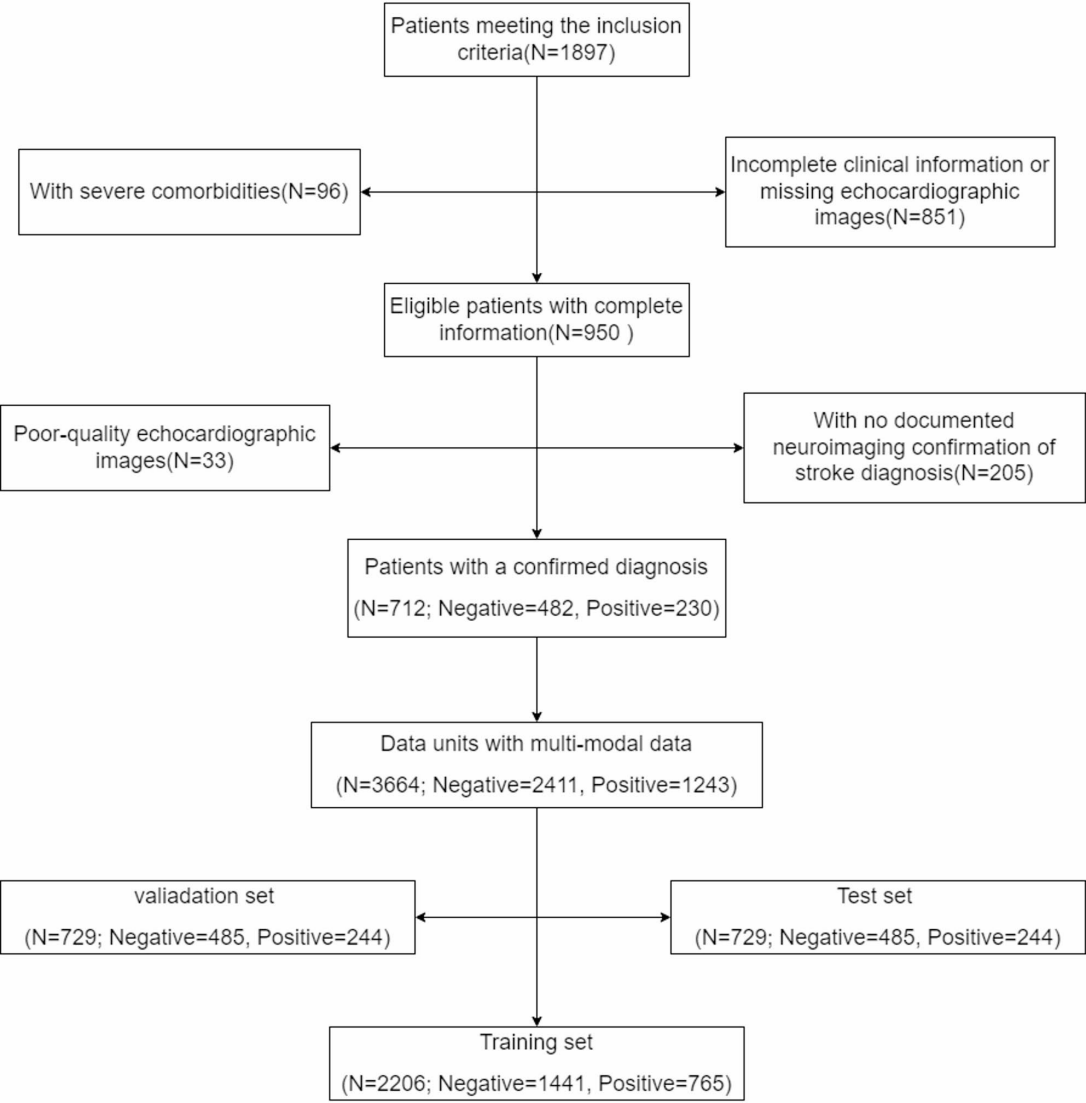
Flowchart of patient selection and dataset creation

As shown in Fig. 1, echocardiographic images from the long-axis, short-axis, and four-chamber views of 712 patients with confirmed diagnoses were processed to produce nine-channel TIFF files. Each file was created by stacking the three echocardiographic views, with each view contributing three channels. For each patient, the number of data units generated ranged from 3 to 9. If a patient had 10 or more images, the highest-quality images were selected to form the final data units. Conversely, patients with 3 or fewer images were considered to have poor-quality echocardiographic images and were excluded from the analysis. Ultimately, 3664 nine-channel TIFF files were integrated with the patients’ corresponding clinical information, including 27 hypertension-related features, to form 3664 data units. Each data unit consists of a combined echocardiographic image and its associated clinical features, providing a robust basis for subsequent model development and analysis.

Although the data units contain repeated clinical information, the data from the same patient were assigned to only one of the test, train, or validation sets to prevent data leakage and reduce the risk of overfitting. Regarding the 27 hypertension-related clinical features, a small portion of these features had missing values (< 10%), which were imputed using mean values to maintain data integrity. For clinical features with more than 10% missing values, they were excluded during the feature collection phase to ensure the robustness and reliability of the dataset. This preprocessing strategy ensures that the echocardiographic images and clinical features provide a clean and consistent input for subsequent model training and evaluation.

**Fig. 2:**
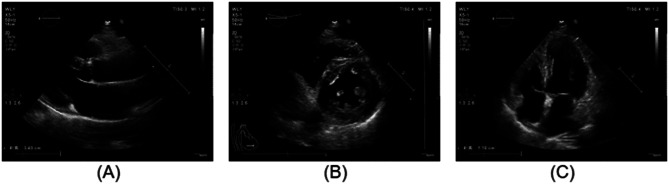
(**A**) The four-chamber view shows all four heart chambers (left/right atria and ventricles), essential for evaluating chamber size, function, and relationships. (**B**) The long-axis view highlights the left ventricle, aorta, and valves (mitral and aortic), aiding in the assessment of ventricular function and valve abnormalities. (**C**) The short-axis view provides cross-sectional images of the heart at various levels, showing structures like papillary muscles and left ventricular wall thickness

Figure 2 presents representative examples of the selected ultrasound images, including the four-chamber, long-axis, and short-axis echocardiographic views. These views were selected because they collectively cover key cardiac structures implicated in cardiogenic stroke risk, including the left atrium, left atrial appendage, left ventricle, and valvular regions—structures commonly associated with thrombus formation or embolic sources in conditions like atrial fibrillation or ventricular dysfunction. Due to data availability constraints, we used static images instead of dynamic video sequences. While real-time imaging is optimal for diagnosing dynamic pathologies like patent foramen ovale, static frames can still capture indirect morphological markers (e.g., LA enlargement, spontaneous echo contrast) that correlate with stroke risk.

Regarding the image formats, our dataset comprised both grayscale B-mode images (primarily for structural assessment) and color Doppler images (for visualizing blood flow, especially when valvular regurgitation or shunts were clinically present). To ensure a uniform input format for our deep learning model, all echocardiographic images were processed and converted into a 3-channel (RGB) TIFF format. This standardization allowed our model to learn comprehensively from both structural information (from B-mode) and hemodynamic information (from color Doppler) when present. To ensure clinical relevance and data quality, two board-certified radiologists (each with ≥ 2 years of echocardiography experience) manually curated the images, selecting diagnostic frames and excluding poor-quality or non-diagnostic ones, irrespective of whether they were B-mode or color Doppler, as long as they contained relevant clinical information.

### Clinical variables

A total of 27 stroke-related risk factors, listed in Table 1, were included in the study. These factors are clinically relevant indicators that can be easily assessed in practice. All risk factors were transformed into variables for model development.

**Table 1:** The risk factors with a definition in this study

Risk factors	Definition
Age	Age of the patients, ranging from 40 years to the maximum observed age.
Gender	Female/Male
Systolic pressure	Systolic blood pressure of the patient, measured in mmHg
Diastolic pressure	Diastolic blood pressure of the patient, measured in mmHg
Hypertension duration	Duration of hypertension in years.
Height	Height of the patient, measured in centimeters (cm)
Weight	Weight of the patient, measured in kilograms (kg)
BMI	Body Mass Index of the patient, calculated as weight (kg) divided by height (m) squared
Drink	Drinking status of the patient, encoded as 0 (non-drinker) or 1 (drinker)
Smoke	Smoking status of the patient, encoded as 0 (non-smoker) or 1 (smoker)
Family history	Presence of family history of stroke, encoded as 0 (no) or 1 (yes)
Blood fat	Blood fat level, encoded (specific encoding not provided)
Blood sugar	Blood sugar level, encoded (specific encoding not provided).
Antihypertensive drug use	A binary indicator (Yes/No) of whether the patient is currently using antihypertensive medications.
COVID19	COVID-19 infection status, encoded as 0 (negative) or 1 (positive).
Cardiac Function Impairment	A binary categorical variable indicating the severity of cardiac functional impairment,
Cardiac Structural Abnormality	categorized based on NYHA classification: • **0**: NYHA Class I or II (normal daily activity unaffected). • **1**: NYHA Class III or IV (daily activity significantly limited).
RWT	Relative wall thickness, a measure used in echocardiography.
LVMI	Left ventricular mass index, a measure used in echocardiography
AAO	Ascending aorta diameter, a measure used in echocardiography
LA	Left atrium diameter, a measure used in echocardiography
LV	Left ventricle diameter, a measure used in echocardiography
IVSD	Interventricular septum diameter, a measure used in echocardiography
LVPW	Left ventricular posterior wall thickness, a measure used in echocardiography
LVEF	Ejection fraction, a measure of the heart’s pumping efficiency, expressed as a percentage
Septal e'	Early diastolic velocity of the septal mitral annulus, a measure used in echocardiography
Lateral e'	Early diastolic velocity of the lateral mitral annulus, a measure used in echocardiography

**Fig. 3:**
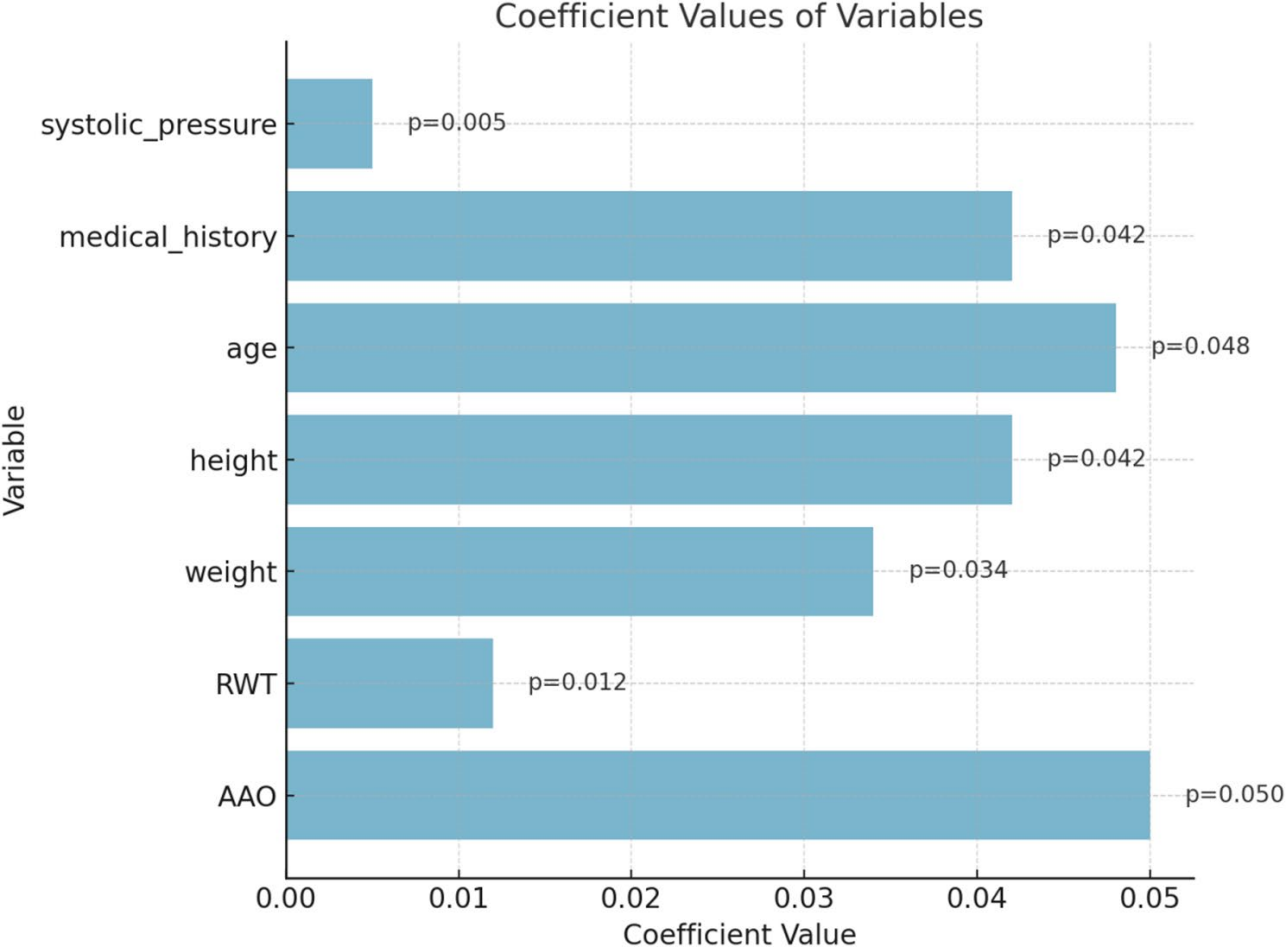
The risk factors in multivariable logistic regression analysis

Risk factors with a P-value < 0.05 in the statistical analysis were considered statistically significant and are listed in Table 2. Continuous variables were analyzed using Student’s t-test, while categorical variables were assessed with the chi-square test. A total of 13 variables were identified as significant. To determine the optimal variables for constructing the prediction model, a multivariable logistic regression analysis was performed, and the results were expressed in terms of P-values. The area under the curve (AUC) was utilized to evaluate the performance and predictive accuracy of the model. Seven variables (Systolic Pressure, Medical History, Age, Height, Weight, RWT, AAO) showed a statistically significant difference (*P* < 0.05) in the multivariable logistic regression analysis. The results of the multivariable logistic regression analysis are displayed as forest plots in Fig. 3.

**Table 2:** Baseline characteristics of the total cohort

Risk Factor	Stroke Mean	Non-Stroke Mean	*P*-Value
Systolic pressure	174.48	164.71	0.01
Diastolic pressure	97.41	97.53	0.938
Medical history	12.03	7.63	0.01
Age	66.78	56.89	0.001
Height	163.24	164.84	0.104
Weight	67.1	69.44	0.128
BMI	25.16	25.45	0.535
Gender	0.52	0.55	0.585
Drink	0.15	0.08	0.051
Smoke	0.3	0.24	0.191
Family history	0.49	0.59	0.065
Blood fat	0.7	0.59	0.045
Blood sugar	0.42	0.3	0.023
Use antihypertensive drugs	0.79	0.68	0.025
COVID19 positive	0.48	0.52	0.469
Function change	0.01	0.01	0.795
Structural change	0.64	0.4	0.001
RWT	0.11	0.04	0.004
LVMI	0.3	0.19	0.013
AAO	0.51	0.28	0.001
LA	0.22	0.33	0.495
LV	0.07	0.04	0.391
IVSD	0.26	0.15	0.01
LVPW	0.03	0.01	0.331
LVEF	0.01	0.01	0.734
Septal e'	0.46	0.31	0.005
Lateral e'	0.58	0.42	0.005

### FPN and EFC modules

Feature Pyramid Networks (FPN) and the EFC module play a critical role in improving feature extraction and fusion for ultrasound imaging. The FPN is designed to effectively integrate multi-scale features, ensuring that both low-level spatial details and high-level semantic information are preserved. However, traditional feature fusion strategies within FPNs often suffer from weak correlations between layers and redundant features, which can hinder the accurate detection of small and complex lesions.

**Fig. 4:**
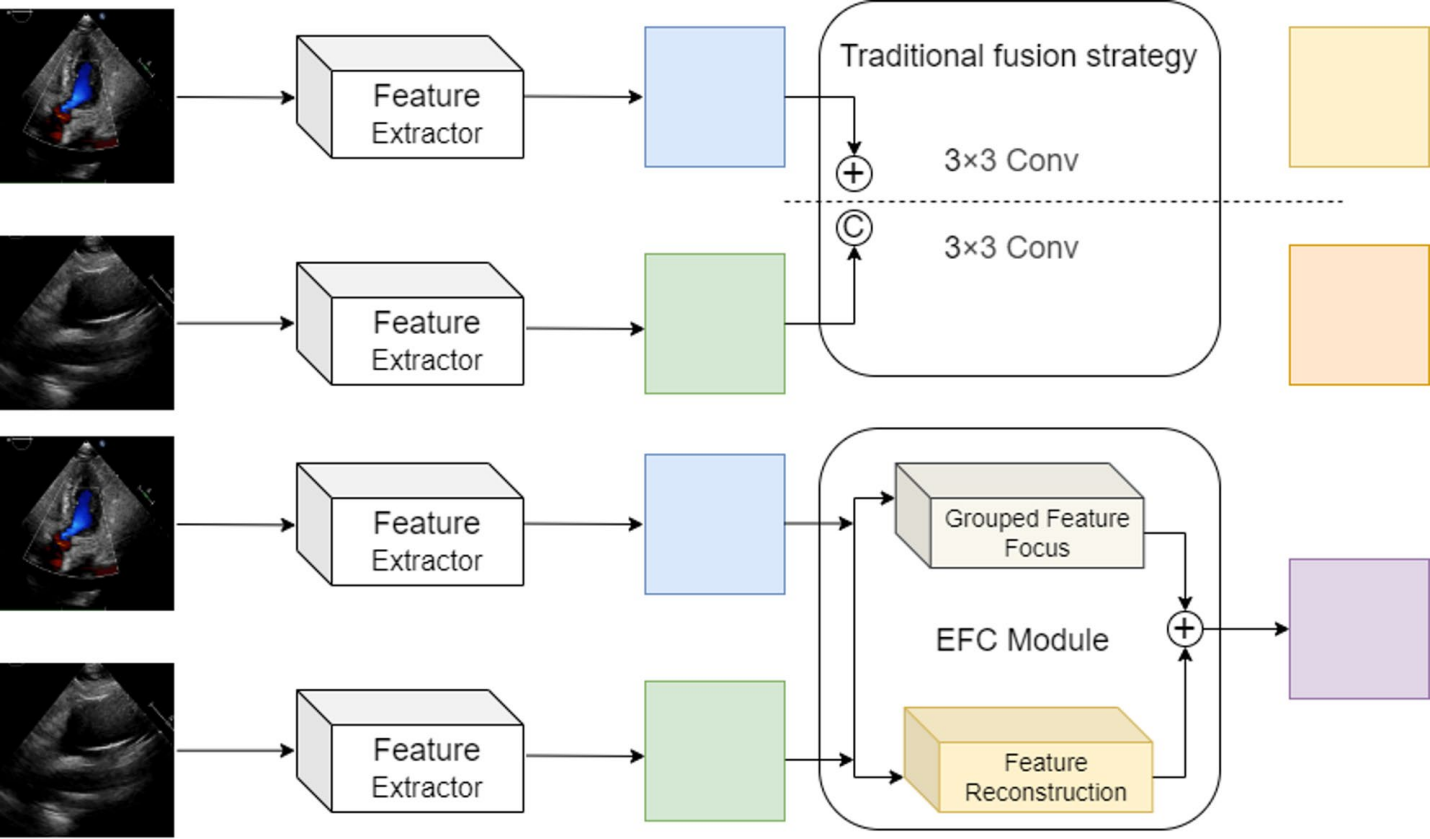
While the traditional method suffers from weak feature correlations and redundancy, the EFC module incorporates Grouped Feature Focus and Feature Reconstruction to enhance feature representation and recover small object details, resulting in improved detection performance

To address these limitations, the EFC module was introduced, as illustrated in Fig. 4. The EFC module consists of two key components: the Grouped Feature Focus (GFF) and Feature Reconstruction (FR). The GFF selectively emphasizes critical feature groups, enhancing the representation of important structures. The FR further refines the fused features, recovering lost information and improving small object detection. Compared to the traditional fusion approach, the EFC module demonstrates superior performance by reducing redundancy and strengthening feature correlations, leading to more accurate lesion detection and classification.

### MSEF module

To further improve upon the EFC module and address the limitations of traditional multi-scale feature fusion strategies, we propose the MSEF module. While EFC effectively enhances feature correlation and reduces redundancy, its performance in handling small targets under complex and dense backgrounds can still be optimized. MSEF builds on EFC by introducing additional mechanisms to strengthen the coordination between deep semantic features and shallow high-resolution features, resulting in more effective multi-scale fusion and improved small target representation.

**Fig. 5:**
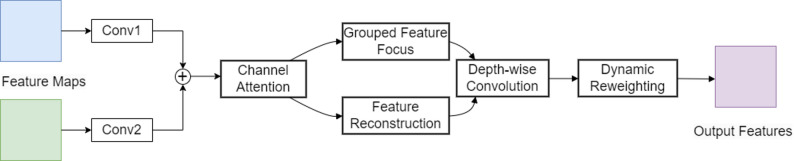
The architecture of the MSEF module. Input feature maps are processed through 1 × 1 convolutions (Conv1 and Conv2) to extract initial representations. Channel Attention selectively enhances important features across channels, while the Grouped Feature Focus refines spatial and grouped information. Simultaneously, Feature Reconstruction recovers lost details and complements the grouped focus. The refined features are further processed by Depth-wise Convolution to extract fine-grained details efficiently, followed by Dynamic Reweighting, which adjusts feature importance dynamically. The final optimized output features are then produced for subsequent network operations

As shown in Fig. 5, MSEF integrates advanced feature processing techniques to enhance feature correlation, minimize redundancy, and maintain computational efficiency. It incorporates four key submodules:

1. Grouped Feature Focus (GFF): This submodule captures contextual information to strengthen the representation of small targets while generating spatial weights to focus on critical regions, ensuring small targets are not overlooked in complex backgrounds. By dividing feature maps into groups based on channels, it enables localized interaction and enhances channel-wise feature correlation, improving feature alignment and representation.
2. Multi-Layer Feature Reconstruction (MFR): MFR separates strong and weak features to avoid interference, ensuring that weak features are independently optimized while preserving the integrity of strong features. It applies fine-grained operations like 1 × 1 convolutions to refine strong features, while using lightweight depth-wise separable convolutions to extract richer information from weak features. The reconstructed features are fused layer by layer, preserving small target details and semantic information for better representation.
3. Channel Attention Submodule: This submodule applies global average pooling to capture global context and uses a 1D convolution to model channel interactions, enhancing the relevance of feature channels. Unlike traditional methods, it avoids dimension reduction, ensuring that critical channel features are preserved, thereby improving overall feature correlation. The mechanisms involved are as follows:

1 \:s=W*z

The above equation represents the operation where the weight *\:W* acts on the input vector *\:z* through a convolution, resulting in a new channel attention representation *\:s*.

2 $$\:{z}_{c}=\frac{1}{H\cdot\:W}{\sum\:}_{i=1}^{H}{\sum\:}_{j=1}^{W}{X}_{c}\left(i,j\right),c=\mathrm{1,2},,C$$

The above equation describes the global average pooling operation applied to each channel $\:\mathrm{c}$ of the input feature map $\:\mathrm{X}$. This computes the global contextual information $\:{\mathrm{z}}_{\mathrm{c}}$ for each channel by averaging all spatial locations.

4. Position Attention Submodule: By applying attention along the horizontal and vertical axes of the feature maps, this submodule extracts spatial structure information through pooling operations. It enables precise focus on key spatial regions, enhancing the model’s ability to locate and represent small targets accurately. The mechanisms involved are as follows:

3 $$\:{P}_{h}\left(i,c\right)=\frac{1}{W}{\sum\:}_{j=1}^{W}{X}_{c}\left(i,j\right)$$

This formula represents the global average pooling operation along the horizontal axis (width $\:\mathrm{W}$) for the $\:\mathrm{c}$-th channel of the input feature map $\:\mathrm{X}$. It aggregates spatial information across the horizontal dimension at height position $\:\mathrm{i}$.

4 $$\:{\widehat{X}}_{c}\left(i,j\right)={\alpha\:}_{h}\left(i,c\right)\cdot\:{X}_{c}\left(i,j\right)+{\alpha\:}_{v}\left(j,c\right)\cdot\:{X}_{c}\left(i,j\right)$$

This formula represents the position attention mechanism for enhancing feature maps. Specifically, it combines the horizontal attention weight $\:{\alpha\:}_{h}\left(i,c\right)$ and the vertical attention weight $\:{{\upalpha\:}}_{\mathrm{v}}\left(\mathrm{j},\mathrm{c}\right)$, which are applied to the $\:\mathrm{c}$-th channel of the input feature map $\:{\mathrm{X}}_{\mathrm{c}}\left(\mathrm{i},\mathrm{j}\right)$. The resulting weighted feature map $\:{\widehat{\mathrm{X}}}_{\mathrm{c}}\left(\mathrm{i},\mathrm{j}\right)$ is obtained by aggregating spatial information along both the horizontal and vertical axes, ensuring that important spatial relationships are captured across the input feature space.

The MSEF module offers several advantages, making it a robust and efficient solution for multi-scale feature fusion. It enhances feature correlation by improving the spatial and semantic alignment of multi-scale features, which significantly strengthens the representation of small targets. The MFR submodule further reduces redundancy through precise feature reconstruction, ensuring that valuable information is retained while minimizing unnecessary fusion. Additionally, its lightweight design optimizes computational efficiency, effectively reducing both parameters and FLOPs without compromising detection accuracy. Moreover, the MSEF module’s plug-and-play compatibility allows seamless integration into various backbone networks, highlighting its versatility and applicability across a wide range of computer vision tasks.

In summary, the MSEF module effectively overcomes the limitations of traditional feature fusion strategies by providing a robust and efficient approach for multi-scale feature integration. Through grouped feature focus, feature reconstruction, and attention mechanisms, it enhances small target representation and delivers superior performance in complex ultrasound images. This makes the MSEF module particularly well-suited for tasks requiring high precision, such as small target detection, segmentation, and classification in cardiac ultrasound imaging.

**Fig. 6:**
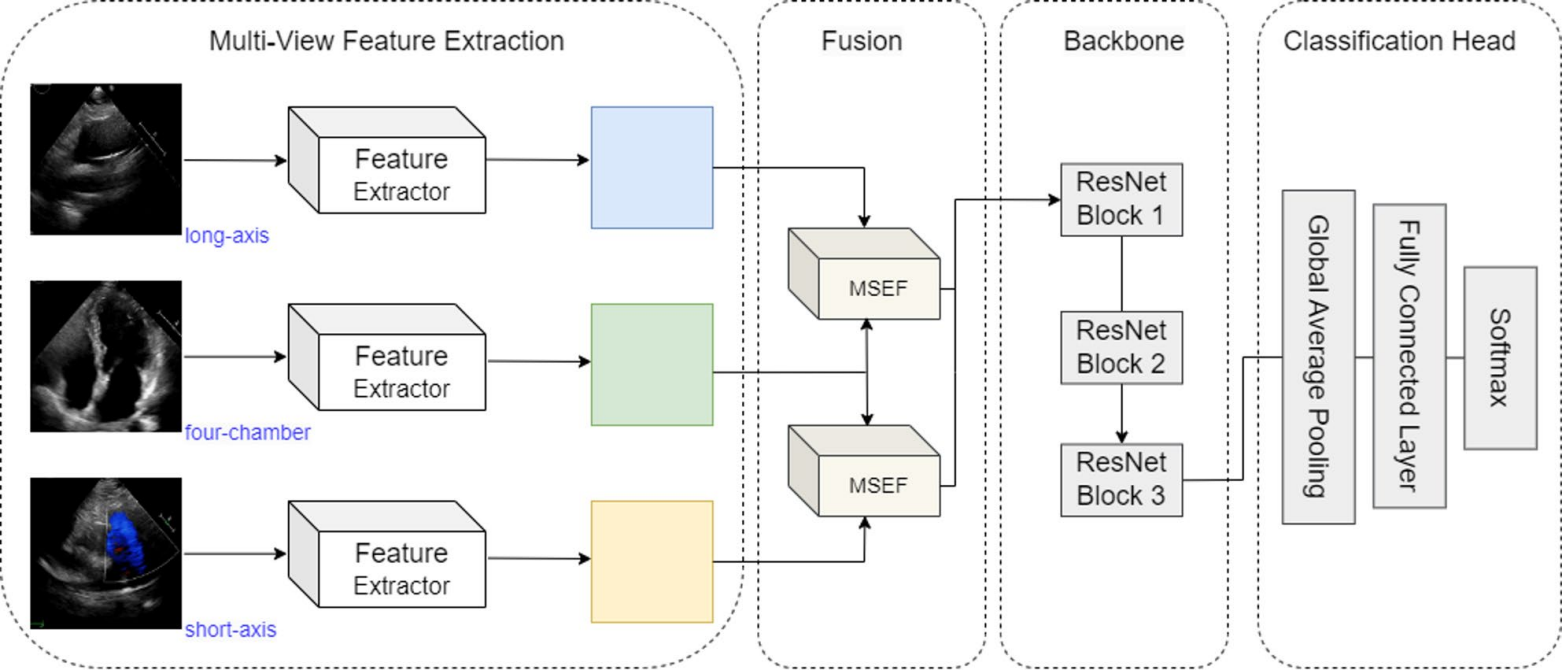
Multi-view echocardiographic image classification pipeline. In the Multi-View Feature Extraction stage, features are extracted independently from three echocardiographic views. These features are then fused to integrate spatial and semantic information. The fused features are passed through a ResNet-based Backbone to further refine the representations. Finally, the Classification Head is applied to reduce the feature dimensions and produce the final classification probabilities

As shown in Fig. 6, the MSEF module plays a crucial role in integrating multi-view echocardiographic features for the prediction of stroke risk. By fusing features extracted from long-axis, four-chamber, and short-axis views, MSEF ensures that spatial and semantic information from multiple perspectives is effectively combined. To further enhance the quality of feature integration, the long-axis and short-axis features are first fused individually with the four-chamber view features, which serves as a baseline due to its comprehensive representation of the heart’s overall structure. This stepwise fusion approach effectively leverages the complementary nature of different echocardiographic views, balancing global structural information with detailed local features while reducing feature redundancy and conflicts. Such a strategy also ensures that nuanced patterns, particularly small targets and subtle abnormalities, are accurately captured. The multi-scale feature integration enabled by MSEF allows the model to better capture subtle variations critical for stroke risk prediction, as these variations are often linked to cardiac structure and function abnormalities. The attention mechanisms within MSEF further emphasize key regions of the images, enabling the model to focus on clinically relevant features while minimizing noise. Overall, the application of MSEF significantly improves the robustness and accuracy of echocardiographic-based stroke risk prediction, addressing the challenges posed by complex and heterogeneous ultrasound data.

### Experimental setup

The code for both EFC and MSEF modules is provided in Supplementary_code.py, and all required Python package versions are listed in Supplementary_version_list.txt. The experiments were conducted using Python 3.11 on an Ubuntu 18.04 operating system.

For network implementation, either PyTorch or the MMPretrain framework can be used. It is necessary to modify the transform functions to accommodate TIFF image inputs, as it have specific channel characteristics. The mean and standard deviation (std) of ultrasound image channels typically hover around 40, but it is recommended to adjust these values based on the specific dataset being used.

The training process was conducted with a batch size of 32, a learning rate of 0.0001, and the SGD (Stochastic Gradient Descent) optimizer. Manual tuning was performed for the learning rate and batch size, initially starting with a learning rate of 0.001 and progressively reducing it to 0.0001 based on validation performance. The model was trained on a single NVIDIA A100 GPU with 80GB memory, with each epoch taking approximately 1.2 min, resulting in a total training time of 2 h over 100 epochs. For devices with limited GPU memory, we recommend proportionally reducing the batch size and learning rate while maintaining their ratio (e.g., halving the batch size to 16 and lowering the learning rate to 0.00005). Alternatively, simplifying the network architecture can help alleviate memory constraints during training.

## Results

### Comparison of fusion module performance

To objectively evaluate the effectiveness of our proposed method, we utilized a set of quantitative metrics, including Accuracy, Precision, Recall, and F1 Score, to ensure a comprehensive performance assessment.

Table 3 presents a summary of the results for three fusion methods—FPN, EFC and MSEF —evaluated on both the validation set and the test set. These metrics provide an in-depth comparison of each method’s performance.

The results clearly demonstrate that the MSEF method is the most robust across both datasets. It consistently achieves the highest Accuracy and F1 Score, showcasing its ability to effectively balance Precision and Recall. While the EFC method achieves strong Precision, its lower Recall reduces its overall performance. In contrast, FPN exhibits moderate performance but struggles to remain competitive, particularly on the test set.

These findings indicate that the multi-scale fusion approach employed by MSEF significantly enhances the model’s generalization capability, resulting in superior performance on unseen data.

**Table 3:** The performance metrics of three methods evaluated on the validation set and the test set

Method	Data Split	Accuracy	Precision	Recall	F1 Score
FPN	Validation Set	74.4%	72.4%	66.7%	67.8%
EFC	Validation Set	73.7%	70.5%	68.2%	69.0%
MSEF	Validation Set	74.6%	71.9%	68.1%	69.1%
FPN	Test Set	70.5%	66.6%	63.0%	63.6%
EFC	Test Set	72.2%	69.7%	63.3%	63.9%
MSEF	Test Set	**76.8%**	**66.2%**	**63.3%**	**64.7%**

### Ablation study of the MSEF module

To gain a deeper understanding of the contribution of each component within the MSEF module, we conducted a systematic ablation study. Specifically, we removed the Channel Attention and Position Attention mechanisms independently to assess their respective roles in the module's overall performance. The results of these experiments, summarized in Table 4, compare three model variants: MSEF without Channel Attention, MSEF without Position Attention, and the Full MSEF Module, evaluated on both the validation set and the test set.

Ablative experiments are crucial for evaluating the effectiveness of individual components within a model, as they provide insights into how specific mechanisms contribute to the overall functionality. The comparative results clearly demonstrate that both Channel Attention and Position Attention play significant roles in enhancing the performance of the MSEF module.

The removal of Channel Attention results in a noticeable drop in Recall on both datasets, with the validation set Recall decreasing to 63.2% and the test set Recall dropping to 69.2%. This reduction indicates that Channel Attention is particularly effective in capturing inter-channel relationships and enhancing feature representations by focusing on the most informative channels. By selectively prioritizing critical channels, this mechanism ensures that the network retains relevant information and suppresses noise, which is particularly important for tasks requiring high sensitivity, as reflected in the Recall metric.

On the other hand, removing Position Attention produces slightly higher Accuracy (77.5% on the validation set) but lowers Precision and F1 Score. The Precision drops to 67.6% on the validation set, highlighting that Position Attention is essential for balancing overall performance. Position Attention focuses on the spatial relationships within the feature maps, ensuring the model captures spatial context effectively. This mechanism is particularly important for preserving structural information, enabling the network to achieve a better balance between Precision and Recall, as evidenced by the higher F1 Score in the Full MSEF Module.

The Full MSEF Module, which integrates both Channel Attention and Position Attention, achieves the best overall performance across all metrics and datasets. On the validation set, it achieves a Recall of 68.1% and an F1 Score of 69.1%, while on the test set, it attains an Accuracy of 76.8% and an F1 Score of 64.7%. This demonstrates that the synergy between the two attention mechanisms enables the model to extract both channel-wise and spatial features effectively, enhancing its ability to generalize to unseen data.

The ablation study underscores the critical importance of the MSEF module's structure, where Channel Attention and Position Attention work collaboratively to address complementary aspects of feature representation. Channel Attention ensures that the model focuses on the most relevant feature channels, improving sensitivity to key patterns, while Position Attention captures spatial dependencies, ensuring that the spatial structure of the data is preserved. By combining these two mechanisms, the Full MSEF Module achieves a superior trade-off between Precision and Recall, leading to robust and reliable performance across diverse datasets. In sum, the ablation study validates the design choices within the MSEF module, demonstrating that the integration of Channel Attention and Position Attention is essential for maximizing the model's overall effectiveness and generalization capability.

**Table 4:** The performance metrics of three model variants evaluated on the validation set and the test set

Model Variant	Data Split	Accuracy	Precision	Recall	F1 Score
MSEF without Channel Attention	Validation Set	72.8%	71.2%	63.2%	63.8%
MSEF without Position Attention	Validation Set	77.5%	67.6%	64.7%	66.1%
Full MSEF Module	Validation Set	74.6%	71.9%	68.1%	69.1%
MSEF without Channel Attention	Test Set	71.0%	68.1%	69.2%	68.5%
MSEF without Position Attention	Test Set	73.5%	70.1%	67.8%	68.6%
Full MSEF Module	Test Set	**76.8%**	**66.2%**	**63.3%**	**64.7%**

### Stroke risk prediction with multimodal integration of clinical and imaging data

Accurate stroke risk prediction relies on effectively integrating clinical data and imaging information, two critical sources of patient information. While imaging data provides detailed anatomical and functional insights, traditional clinical features such as age, blood pressure, cholesterol levels, and comorbidities remain cornerstone predictors for stroke risk, as supported by extensive clinical studies. Leveraging these well-established clinical indicators alongside advanced imaging features ensures a robust and clinically interpretable prediction model.

Our analysis encompassed two primary modeling approaches: models using only clinical features and multimodal models integrating both clinical and imaging data. Table 5 presents the performance metrics for three prediction models (Logistic Regression, Random Forest, and XGBoost) trained exclusively on clinical features, evaluated on both the validation and test sets. Conversely, Table 6 details the superior performance of their multimodal counterparts, which additionally incorporate imaging features extracted from the MSEF module.

**Table 5:** The performance metrics of three prediction models using clinical features only (LR: Logistic Regression, RF: Random Forest, XGB: XGBoost)

Method	Data Split	Accuracy	Precision	Recall	F1 Score
LR	Validation Set	70.60%	54.80%	70.10%	61.50%
RF	Validation Set	71.60%	55.50%	76.20%	64.20%
XGB	Validation Set	74.10%	59.00%	74.20%	65.70%
LR	Test Set	70.20%	54.20%	70.90%	61.50%
RF	Test Set	72.00%	56.50%	71.70%	63.20%
XGB	Test Set	69.40%	53.40%	68.40%	60.00%

**Table 6:** The performance metrics of three multimodal prediction models (LR: Logistic Regression, RF: Random Forest, XGB: XGBoost)

Method	Data Split	Accuracy	Precision	Recall	F1 Score
LR	Validation Set	80.5%	74.0%	71.4%	72.7%
RF	Validation Set	80.7%	74.4%	71.8%	73.0%
XGB	Validation Set	81.1%	74.8%	72.3%	73.6%
LR	Test Set	79.9%	72.8%	70.1%	71.4%
RF	Test Set	80.1%	73.1%	70.5%	71.8%
XGB	Test Set	80.2%	73.4%	70.8%	72.1%

**Fig. 7:**
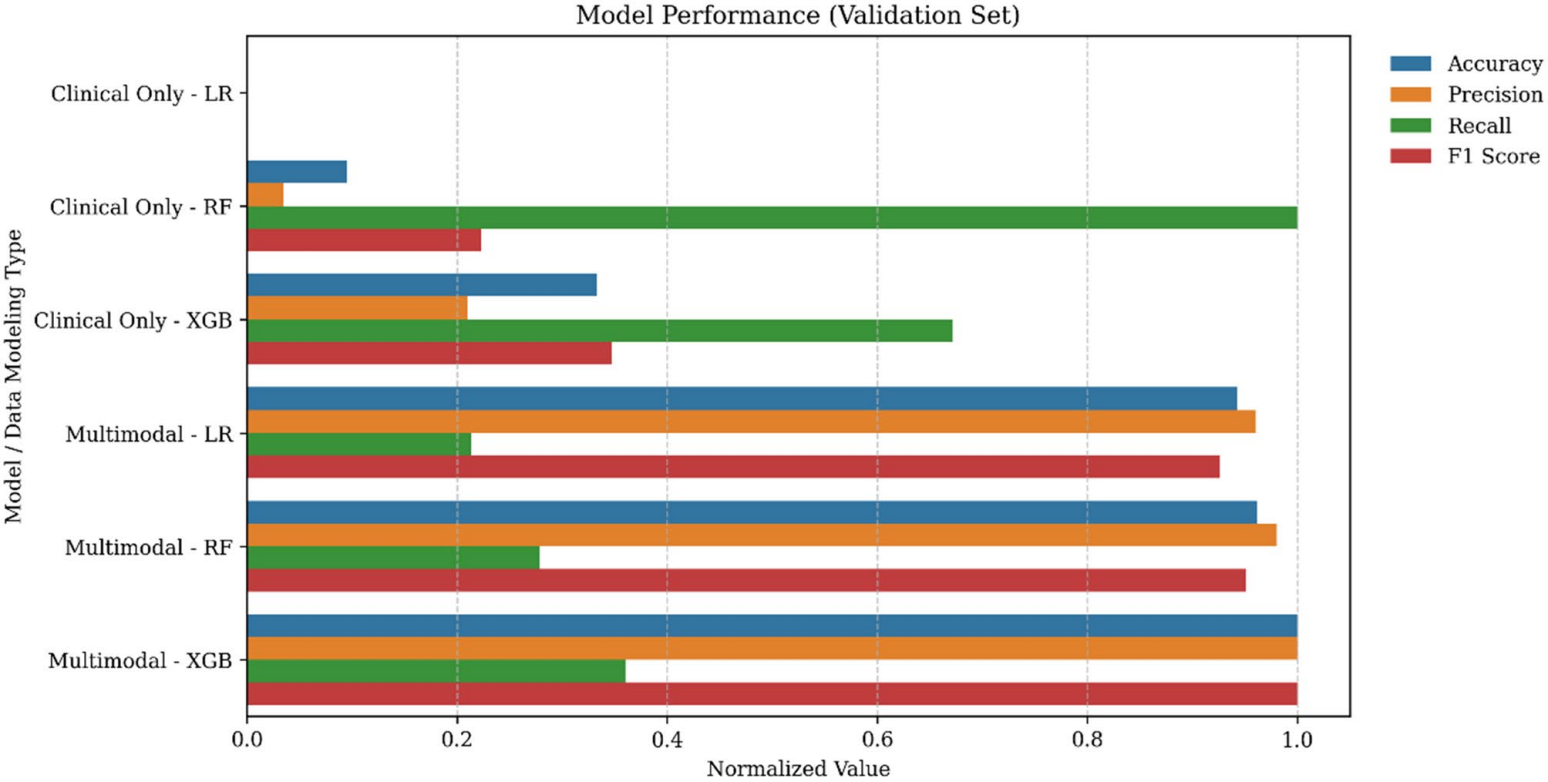
Model performance on validation set

**Fig. 8:**
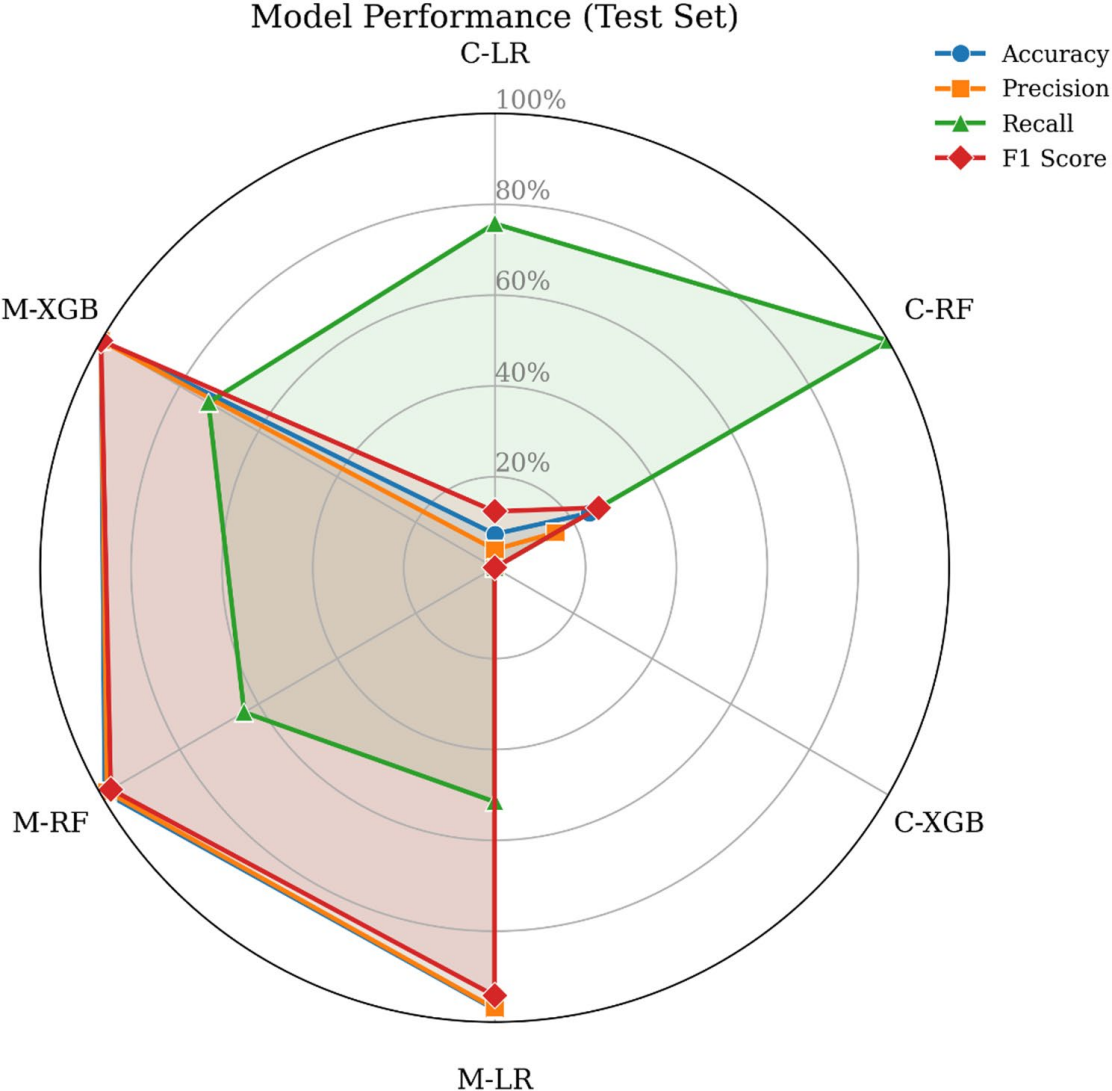
Model performance on test set. The prefix “C” denotes “Clinical Only” models. Conversely, the prefix “M” signifies “Multimodal” models

Visual summaries of these performances are provided in Fig. 7 for the validation set and Fig. 8 for the test set. As depicted in Fig. 7, multimodal models consistently outperform clinical-only models across all metrics (Accuracy, Precision, Recall, and F1 Score) on the validation set, demonstrating the significant value added by imaging data. Figure 8 further corroborates these findings on the test set, highlighting the robust and generalized improvement achieved through multimodal integration, with Multimodal-XGB (M-XGB) showing particularly strong and balanced performance.

The integration of traditional clinical features with imaging-derived data is rooted in established clinical practice and research. Clinical features such as age, hypertension, diabetes, and lifestyle factors have been extensively validated as independent stroke predictors in prior studies. Imaging features, particularly those extracted through advanced fusion methods like MSEF, capture subtle and spatially distributed changes that are often invisible to manual inspection.

By combining these two modalities, the models benefit from:

1. Enhanced Predictive Power: Clinical features provide a solid baseline prediction, while imaging features refine the model's ability to capture complex patterns indicative of stroke risk.
2. Clinical Interpretability: Traditional clinical predictors offer transparency and trust, enabling clinicians to validate and interpret the model's output more easily.
3. Generalizability: The inclusion of clinical data stabilizes the model's performance across datasets, reducing overfitting to imaging-specific noise.

In sum, the results demonstrate that integrating clinical features with imaging-derived data significantly enhances stroke risk prediction performance. Among the evaluated models, XGBoost consistently achieves the best results, underscoring its ability to handle multimodal data effectively. This multimodal integration approach aligns with prior research and clinical practice, highlighting the importance of leveraging well-established clinical predictors alongside advanced imaging features to achieve reliable, interpretable, and clinically applicable stroke prediction models.

## Discussion

In this study, we proposed a novel stroke risk prediction framework that integrates clinical indicators with echocardiographic imaging features using a Multi-Scale Effective Fusion module. By combining traditional clinical factors and advanced imaging analysis, we achieved enhanced performance and demonstrated the importance of multimodal data integration for stroke risk assessment.

### Significance of integrating clinical and imaging data

**Fig. 9:**
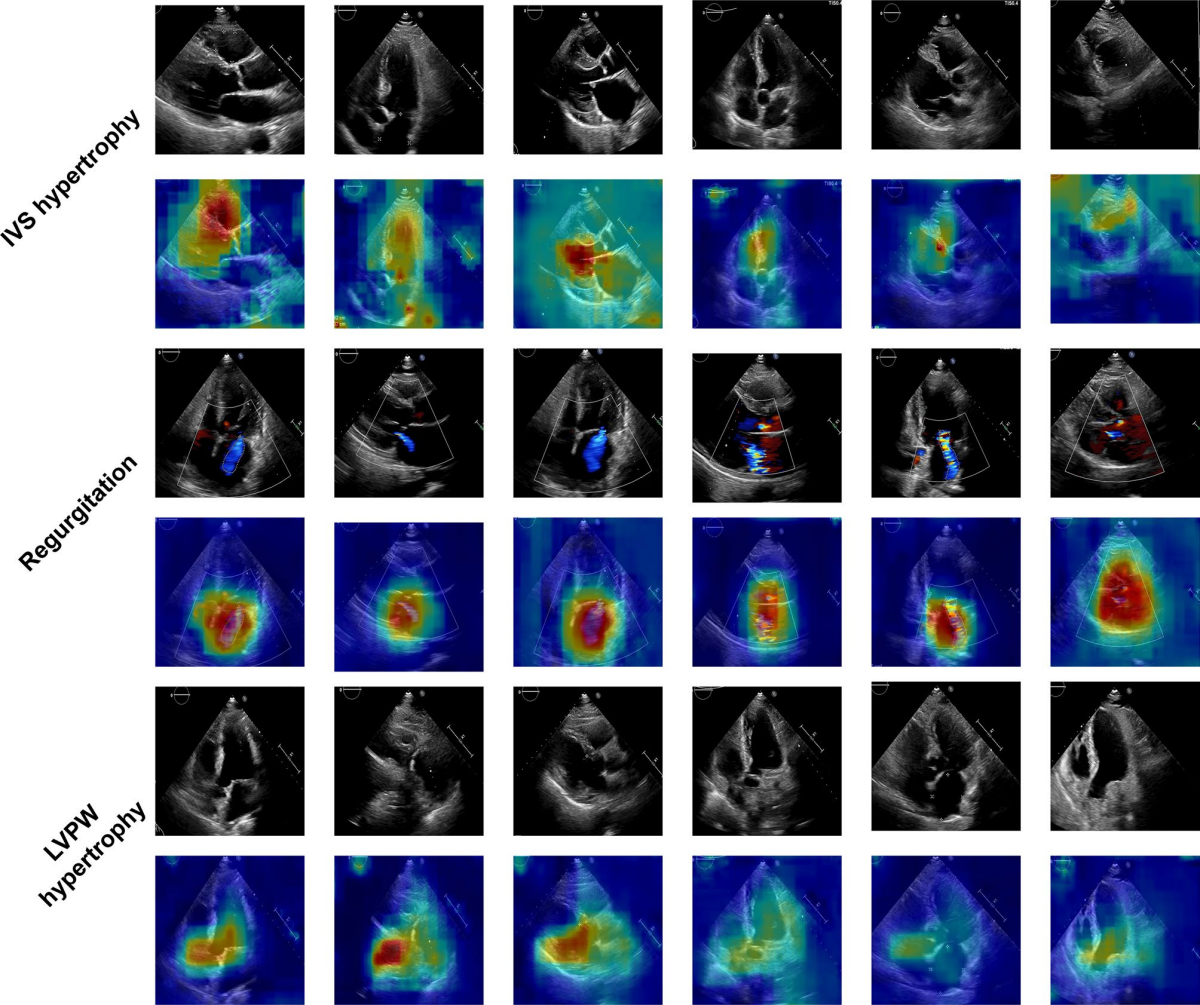
Model attention heatmaps on echocardiographic images for stroke risk prediction. This figure visually demonstrates the regions of highest model attention when assessing stroke risk. Rows 1, 3, and 5 display original echocardiographic images (B-mode and Color Doppler). Rows 2, 4, and 6 show the corresponding images with overlaid Grad-CAM heatmaps, where warm colors (red/orange) indicate high attention and cool colors (blue/green) indicate lower attention. The heatmaps consistently highlight critical cardiac features associated with stroke risk, such as interventricular septal thickening, left ventricular posterior wall thickening, left atrial chamber morphology, and valvular regurgitation

Traditional stroke risk models primarily rely on clinical indicators, such as blood pressure, age, and medical history, which have long been validated as strong predictors in clinical practice [29–31]. However, these models often fall short in accounting for subtle cardiac morphological and functional changes detectable through imaging. Echocardiographic data, including long-axis, short-axis, and four-chamber views, offer critical insights into cardiac structure and function, such as valve abnormalities, wall motion dynamics, and atrial enlargement, which are pivotal for stroke pathogenesis. Integrating these imaging-derived features with clinical data bridges the gap between visual assessments and quantitative predictions, leading to a more comprehensive and interpretable risk prediction model. As shown in Section “Stroke risk prediction with multimodal integration of clinical and imaging data”, the multimodal integration approach demonstrated superior performance compared to single-modality methods, a trend clearly corroborated by the performance visualizations in Fig. 9.

Figure 9 visually demonstrates the regions of highest model attention when assessing stroke risk. Specifically, we can observe that the highly activated regions in the heatmaps frequently converge on critical cardiac features such as:

1. Myocardial Wall Thickness and Cardiac Chamber Morphology: The heatmaps prominently highlight the myocardial walls of the ventricles, specifically the interventricular septum and the left ventricular posterior wall, along with the left atrial chamber. The model’s intense focus on these areas suggests it is adept at discerning features such as interventricular septal thickening (or hypertrophy) and left ventricular posterior wall thickening (LVPW thickening). Both forms of left ventricular hypertrophy (encompassing these wall thickenings) and left atrial enlargement are well-established independent risk factors for stroke due to their association with increased cardiac workload, diastolic dysfunction, and a heightened propensity for arrhythmias (like atrial fibrillation) and thrombus formation. This indicates the model is effectively capturing critical changes in myocardial structure.
2. Cardiac Valve Regions and Valvular Regurgitation: The heatmaps consistently emphasize the cardiac valves (e.g., mitral, aortic, tricuspid) and their surrounding areas. This focus is frequently coupled with strong activations in areas depicting valvular regurgitation (the abnormal backward flow of blood through a valve) on both B-mode and color Doppler images. Significant valvular regurgitation (e.g., mitral regurgitation, aortic regurgitation, or tricuspid regurgitation) can lead to chronic volume overload, subsequent chamber dilation and remodeling, and an increased risk of atrial fibrillation and subsequent cardioembolic events. The model appears to interpret the presence and severity of these regurgitant jets as crucial indicators impacting stroke risk.
3. Complex Hemodynamics: Beyond the direct assessment of structural thickening and valvular integrity, the model also demonstrates an ability to interpret more complex and dynamic hemodynamic information. While partly captured by valvular regurgitation, the heatmaps also suggest attention to subtle alterations in blood flow patterns (e.g., turbulence, subtle shunts) which, though not always visually obvious, can contribute to overall cardiovascular strain and stroke risk.

This highlights the advantages of advanced machine learning algorithms in handling complex, heterogeneous data, where clinical and imaging features complement one another to refine predictions.

### The role of the MSEF module

The MSEF module, introduced in this study, played a critical role in improving feature extraction from echocardiographic images. Previous studies have demonstrated that multi-scale feature fusion enhances representation learning, especially for subtle or small abnormalities. Our ablation study in Section “Ablation study of the MSEF module” revealed that both Channel Attention and Position Attention mechanisms significantly contribute to feature enhancement. Channel Attention prioritizes critical channels, improving the detection of informative patterns, while Position Attention captures spatial relationships, enabling the model to preserve structural details. By combining these mechanisms, the MSEF module effectively addresses the challenges posed by heterogeneous echocardiographic data, ensuring robust and accurate feature integration. The superior performance of the Full MSEF Module compared to its ablated variants underscores the necessity of a multi-dimensional attention strategy. Removing either Channel or Position Attention led to performance degradation, confirming that the combination of both mechanisms achieves optimal results for stroke risk prediction.

### Clinical implications

Our findings align with existing clinical knowledge, emphasizing the continued relevance of traditional clinical features while leveraging advancements in imaging and machine learning. Clinical predictors such as age, blood pressure, and medical history remain highly interpretable and trusted by clinicians. Integrating these indicators with echocardiographic features enhances predictive power while ensuring the model’s clinical applicability and transparency. This multimodal framework allows for the early identification of stroke risk, enabling timely preventive interventions and personalized treatment strategies for at-risk populations.

Furthermore, the success of the MSEF-based approach highlights the potential of deep learning models in clinical settings. The ability to analyze echocardiographic data at a granular level enables the detection of subtle cardiac abnormalities, such as early atrial enlargement or wall motion irregularities, which may be overlooked during manual interpretation. Combined with clinical factors, these insights provide a more holistic understanding of stroke risk.

### Limitations and future work

Despite its promising results, this study has some limitations. First, the retrospective nature of the dataset may introduce selection bias, and future studies should validate the proposed framework using prospective data. Second, our reliance on static echocardiographic images, while clinically interpretable for structural abnormalities (e.g., left atrial enlargement), may limit sensitivity for dynamic pathologies such as PFO detection, which typically requires real-time imaging. Future work will incorporate dynamic video sequences to capture transient hemodynamic features. Additionally, incorporating other imaging modalities, such as cardiac MRI, may provide additional insights and further improve predictive accuracy. Future work will focus on expanding the dataset to include diverse patient populations and exploring other fusion strategies for multimodal data integration.

## Conclusions

In conclusion, this study demonstrates the importance of integrating clinical indicators with advanced imaging-derived features for stroke risk prediction. The proposed MSEF module effectively captures multi-scale echocardiographic information, improving predictive accuracy and robustness. By leveraging the strengths of both clinical data and machine learning-based imaging analysis, our approach provides a clinically interpretable and highly accurate tool for early stroke risk assessment, paving the way for improved prevention and personalized interventions.

## Supplementary Information

Below is the link to the electronic supplementary material.

Supplementary Material 1

Supplementary Material 2

## Data Availability

The datasets are not publicly available due to patient privacy restrictions but are available from the corresponding author on reasonable request.

## Abbreviations

US: Ultrasound
MSEF: Multi-scale effective fusion
GFF: Global Feature Fusion
MFR: Multi-Feature Reconstruction
PFO: Patent foramen ovale
FPN: Feature Pyramid Networks
AUC: Area under the curve
RWT: Relative wall thickness
LVMI: Left ventricular mass index
AAO: Ascending aorta diameter
LA: Left atrium diameter
LV: Left ventricle diameter
IVSD: Interventricular septum diameter
LVPW: Left ventricular posterior wall thickness
EF: Ejection fraction
