# Supplementary material for: Cardiac echocardiographic analysis with multi-scale effective fusion module: a novel stroke prediction approach
Source: BMC Med Imaging. 2026 May 28;26:370. doi: 10.1186/s12880-026-02460-7 (PMC13411767; doi:10.1186/s12880-026-02460-7)
Supplement: Supplementary file 1 — Supplementary Material 1 [file 12880_2026_2460_MOESM1_ESM.docx]

import math

import torch

import torch.nn as nn

import torch.nn.functional as F

class channel_att(nn.Module):

def __init__(self, channel, b=1, gamma=2):

super(channel_att, self).__init__()

kernel_size = int(abs((math.log(channel, 2) + b) / gamma))

kernel_size = kernel_size if kernel_size % 2 else kernel_size + 1

self.avg_pool = nn.AdaptiveAvgPool2d(1)

self.conv = nn.Conv1d(1, 1, kernel_size=kernel_size, padding=(kernel_size - 1) // 2, bias=False)

self.sigmoid = nn.Sigmoid()

def forward(self, x):

y = self.avg_pool(x)

y = y.squeeze(-1)

y = y.transpose(-1, -2)

y = self.conv(y).transpose(-1, -2).unsqueeze(-1)

y = self.sigmoid(y)

return x * y.expand_as(x)

class local_att(nn.Module):

def __init__(self, channel, reduction=16):

super(local_att, self).__init__()

self.conv_1x1 = nn.Conv2d(in_channels=channel, out_channels=channel // reduction, kernel_size=1, stride=1,

bias=False)

self.relu = nn.ReLU()

self.bn = nn.BatchNorm2d(channel // reduction)

self.F_h = nn.Conv2d(in_channels=channel // reduction, out_channels=channel, kernel_size=1, stride=1,

bias=False)

self.F_w = nn.Conv2d(in_channels=channel // reduction, out_channels=channel, kernel_size=1, stride=1,

bias=False)

self.sigmoid_h = nn.Sigmoid()

self.sigmoid_w = nn.Sigmoid()

def forward(self, x):

_, _, h, w = x.size()

x_h = torch.mean(x, dim=3, keepdim=True).permute(0, 1, 3, 2)

x_w = torch.mean(x, dim=2, keepdim=True)

x_cat_conv_relu = self.relu(self.bn(self.conv_1x1(torch.cat((x_h, x_w), 3))))

x_cat_conv_split_h, x_cat_conv_split_w = x_cat_conv_relu.split([h, w], 3)

s_h = self.sigmoid_h(self.F_h(x_cat_conv_split_h.permute(0, 1, 3, 2)))

s_w = self.sigmoid_w(self.F_w(x_cat_conv_split_w))

out = x * s_h.expand_as(x) * s_w.expand_as(x)

return out

class EFC(nn.Module):

def __init__(self, c1, c2):

super().__init__()

self.conv1 = nn.Conv2d(c1, c2, kernel_size=1, stride=1)

self.conv2 = nn.Conv2d(c2, c2, kernel_size=1, stride=1)

self.conv4 = nn.Conv2d(c2, c2, kernel_size=1, stride=1)

self.bn = nn.BatchNorm2d(c2)

self.sigomid = nn.Sigmoid()

self.group_num = 16

self.eps = 1e-10

self.gamma = nn.Parameter(torch.randn(c2, 1, 1))

self.beta = nn.Parameter(torch.zeros(c2, 1, 1))

self.gate_genator = nn.Sequential(

nn.AdaptiveAvgPool2d((1, 1)),

nn.Conv2d(c2, c2, 1, 1),

nn.ReLU(True),

nn.Softmax(dim=1),

)

self.dwconv = nn.Conv2d(c2, c2, kernel_size=3, stride=1, padding=1, groups=c2)

self.conv3 = nn.Conv2d(c2, c2, kernel_size=1, stride=1)

self.Apt = nn.AdaptiveAvgPool2d(1)

self.one = c2

self.two = c2

self.conv4_gobal = nn.Conv2d(c2, 1, kernel_size=1, stride=1)

for group_id in range(0, 4):

self.interact = nn.Conv2d(c2 // 4, c2 // 4, 1, 1, )

def forward(self, x1, x2):

global_conv1 = self.conv1(x1)

bn_x = self.bn(global_conv1)

weight_1 = self.sigomid(bn_x)

global_conv2 = self.conv2(x2)

bn_x2 = self.bn(global_conv2)

weight_2 = self.sigomid(bn_x2)

X_GOBAL = global_conv1 + global_conv2

x_conv4 = self.conv4_gobal(X_GOBAL)

X_4_sigmoid = self.sigomid(x_conv4)

X_ = X_4_sigmoid * X_GOBAL

X_ = X_.chunk(4, dim=1)

out = []

for group_id in range(0, 4):

out_1 = self.interact(X_[group_id])

N, C, H, W = out_1.size()

x_1_map = out_1.reshape(N, 1, -1)

mean_1 = x_1_map.mean(dim=2, keepdim=True)

x_1_av = x_1_map / mean_1

x_2_2 = F.softmax(x_1_av, dim=1)

x1 = x_2_2.reshape(N, C, H, W)

x1 = X_[group_id] * x1

out.append(x1)

out = torch.cat([out[0], out[1], out[2], out[3]], dim=1)

N, C, H, W = out.size()

x_add_1 = out.reshape(N, self.group_num, -1)

N, C, H, W = X_GOBAL.size()

x_shape_1 = X_GOBAL.reshape(N, self.group_num, -1)

mean_1 = x_shape_1.mean(dim=2, keepdim=True)

std_1 = x_shape_1.std(dim=2, keepdim=True)

x_guiyi = (x_add_1 - mean_1) / (std_1 + self.eps)

x_guiyi_1 = x_guiyi.reshape(N, C, H, W)

x_gui = (x_guiyi_1 * self.gamma + self.beta)

weight_x3 = self.Apt(X_GOBAL)

reweights = self.sigomid(weight_x3)

x_up_1 = reweights >= weight_1

x_low_1 = reweights < weight_1

x_up_2 = reweights >= weight_2

x_low_2 = reweights < weight_2

x_up = x_up_1 * X_GOBAL + x_up_2 * X_GOBAL

x_low = x_low_1 * X_GOBAL + x_low_2 * X_GOBAL

x11_up_dwc = self.dwconv(x_low)

x11_up_dwc = self.conv3(x11_up_dwc)

x_so = self.gate_genator(x_low)

x11_up_dwc = x11_up_dwc * x_so

x22_low_pw = self.conv4(x_up)

xL = x11_up_dwc + x22_low_pw

xL = xL + x_gui

return xL

class MSEF(nn.Module):

def __init__(self, c1, c2):

super().__init__()

self.channel_att = channel_att(c2)

self.local_att = local_att(c2)

self.conv1 = nn.Conv2d(c1, c2, kernel_size=1, stride=1)

self.conv2 = nn.Conv2d(c2, c2, kernel_size=1, stride=1)

self.conv4 = nn.Conv2d(c2, c2, kernel_size=1, stride=1)

self.bn = nn.BatchNorm2d(c2)

self.sigomid = nn.Sigmoid()

self.group_num = 16

self.eps = 1e-10

self.gamma = nn.Parameter(torch.randn(c2, 1, 1))

self.beta = nn.Parameter(torch.zeros(c2, 1, 1))

self.gate_genator = nn.Sequential(

nn.AdaptiveAvgPool2d((1, 1)),

nn.Conv2d(c2, c2, 1, 1),

nn.ReLU(True),

nn.Softmax(dim=1),

)

self.dwconv = nn.Conv2d(c2, c2, kernel_size=3, stride=1, padding=1, groups=c2)

self.conv3 = nn.Conv2d(c2, c2, kernel_size=1, stride=1)

self.Apt = nn.AdaptiveAvgPool2d(1)

self.one = c2

self.two = c2

self.conv4_gobal = nn.Conv2d(c2, 1, kernel_size=1, stride=1)

for group_id in range(0, 4):

self.interact = nn.Conv2d(c2 // 4, c2 // 4, 1, 1, )

def forward(self, x1, x2):

global_conv1 = self.conv1(x1)

bn_x = self.bn(global_conv1)

weight_1 = self.sigomid(bn_x)

global_conv2 = self.conv2(x2)

bn_x2 = self.bn(global_conv2)

weight_2 = self.sigomid(bn_x2)

X_GOBAL = global_conv1 + global_conv2

temp = self.channel_att(X_GOBAL)

x_conv4 = self.conv4_gobal(X_GOBAL)

X_4_sigmoid = self.sigomid(x_conv4)

X_ = X_4_sigmoid * X_GOBAL

X_ = X_.chunk(4, dim=1)

out = []

for group_id in range(0, 4):

out_1 = self.interact(X_[group_id])

N, C, H, W = out_1.size()

x_1_map = out_1.reshape(N, 1, -1)

mean_1 = x_1_map.mean(dim=2, keepdim=True)

x_1_av = x_1_map / mean_1

x_2_2 = F.softmax(x_1_av, dim=1)

x1 = x_2_2.reshape(N, C, H, W)

x1 = X_[group_id] * x1

out.append(x1)

out = torch.cat([out[0], out[1], out[2], out[3]], dim=1)

N, C, H, W = out.size()

x_add_1 = out.reshape(N, self.group_num, -1)

N, C, H, W = X_GOBAL.size()

x_shape_1 = X_GOBAL.reshape(N, self.group_num, -1)

mean_1 = x_shape_1.mean(dim=2, keepdim=True)

std_1 = x_shape_1.std(dim=2, keepdim=True)

x_guiyi = (x_add_1 - mean_1) / (std_1 + self.eps)

x_guiyi_1 = x_guiyi.reshape(N, C, H, W)

x_gui = (x_guiyi_1 * self.gamma + self.beta)

weight_x3 = self.Apt(X_GOBAL)

reweights = self.sigomid(weight_x3)

x_up_1 = reweights >= weight_1

x_low_1 = reweights < weight_1

x_up_2 = reweights >= weight_2

x_low_2 = reweights < weight_2

x_up = x_up_1 * X_GOBAL + x_up_2 * X_GOBAL

x_low = x_low_1 * X_GOBAL + x_low_2 * X_GOBAL

x11_up_dwc = self.dwconv(x_low)

x11_up_dwc = self.conv3(x11_up_dwc)

x_so = self.gate_genator(x_low)

x11_up_dwc = x11_up_dwc * x_so

x22_low_pw = self.conv4(x_up)

xL = x11_up_dwc + x22_low_pw

xL = xL + x_gui + temp

out = self.local_att(xL)

return out

# test code

if __name__ == '__main__':

input1 = torch.randn(1, 32, 64, 64)

input2 = torch.randn(1, 64, 64, 64)

# EFC

# initial

EFC_module = EFC(c1=32,c2=64) #channels of input1 and input2

output =EFC_module(input1,input2)

# output shape

print("EFC_input_shape：", input2.shape)

print("EFC_output_shape：", output.shape)

# MSEF

MSEF_module = MSEF(c1=32, c2=64)

output = MSEF_module(input1, input2)

print("MSEF_input_shape：", input2.shape)

print("MSEF_output_shape：", output.shape)
