# Supplementary material for: Cardiac echocardiographic analysis with multi-scale effective fusion module: a novel stroke prediction approach
Source: BMC Med Imaging. 2026 May 28;26:370. doi: 10.1186/s12880-026-02460-7 (PMC13411767; doi:10.1186/s12880-026-02460-7)
Supplement: Supplementary file 2 — Supplementary Material 2 [file 12880_2026_2460_MOESM2_ESM.docx]

Package Version Editable project location

------------------------ ---------------- ---------------------------------------

absl-py 2.1.0

accelerate 0.26.1

addict 2.4.0

aiofiles 23.2.1

aiohappyeyeballs 2.4.3

aiohttp 3.10.9

aiosignal 1.3.1

albucore 0.0.17

albumentations 1.3.1

aliyun-python-sdk-core 2.15.2

aliyun-python-sdk-kms 2.16.5

annotated-types 0.7.0

anyio 4.6.0

asttokens 2.4.1

attrs 24.2.0

av 13.1.0

basicsr 1.4.2

certifi 2024.8.30

cffi 1.17.1

charset-normalizer 3.3.2

click 8.1.7

clip 0.2.0

colorama 0.4.6

comm 0.2.2

contourpy 1.3.0

controlnet-aux 0.0.9

crcmod 1.7

cryptography 43.0.1

cycler 0.12.1

Cython 3.0.11

debugpy 1.8.6

decorator 5.1.1

diffusers 0.23.1

einops 0.8.0

eval_type_backport 0.2.0

executing 2.1.0

face-alignment 1.3.4

facexlib 0.3.0

fastapi 0.115.0

ffmpy 0.4.0

filelock 3.14.0

filterpy 1.4.5

flatbuffers 24.3.25

fonttools 4.54.1

frozenlist 1.4.1

fsspec 2024.9.0

future 1.0.0

gradio 5.0.1

gradio_client 1.4.0

grpcio 1.66.2

h11 0.14.0

httpcore 1.0.6

httpx 0.27.2

huggingface-hub 0.25.2

idna 3.10

imageio 2.35.1

importlib_metadata 8.5.0

ipykernel 6.29.5

ipython 8.28.0

jax 0.4.34

jaxlib 0.4.34

jedi 0.19.1

Jinja2 3.1.4

jmespath 0.10.0

joblib 1.4.2

jupyter_client 8.6.3

jupyter_core 5.7.2

kiwisolver 1.4.7

lazy_loader 0.4

lightning 2.4.0

lightning-utilities 0.11.7

llvmlite 0.43.0

lmdb 1.5.1

lpips 0.1.4

Markdown 3.7

markdown-it-py 3.0.0

MarkupSafe 2.1.5

mat4py 0.6.0

matplotlib 3.9.2

matplotlib-inline 0.1.7

maturin 1.7.4

mdurl 0.1.2

mediapipe 0.10.15

ml_dtypes 0.5.0

mmagic 1.2.0.dev0 /chenyan123/models/mmagic/mmagic

mmcv 2.0.0

mmdet 3.3.0 /chenyan123/models/mmdet/mmdetection

mmengine 0.10.5

mmpretrain 1.2.0 /chenyan123/models/mmpre/mmpretrain

mmsegmentation 1.1.2 /chenyan123/models/mmseg/mmsegmentation

mmyolo 0.6.0 /chenyan123/models/mmdet/mmyolo

model-index 0.1.11

modelindex 0.0.2

mpmath 1.3.0

multidict 6.1.0

nest-asyncio 1.6.0

networkx 3.3

ninja 1.11.1.1

numba 0.60.0

numpy 1.26.4

nvidia-cublas-cu12 12.4.2.65

nvidia-cuda-cupti-cu12 12.4.99

nvidia-cuda-nvrtc-cu12 12.4.99

nvidia-cuda-runtime-cu12 12.4.99

nvidia-cudnn-cu12 9.1.0.70

nvidia-cufft-cu12 11.2.0.44

nvidia-curand-cu12 10.3.5.119

nvidia-cusolver-cu12 11.6.0.99

nvidia-cusparse-cu12 12.3.0.142

nvidia-nccl-cu12 2.20.5

nvidia-nvjitlink-cu12 12.4.99

nvidia-nvtx-cu12 12.4.99

opencv-contrib-python 4.10.0.84

opencv-python 4.10.0.84

opencv-python-headless 4.10.0.84

opendatalab 0.0.10

openmim 0.3.9

openxlab 0.1.1

opt_einsum 3.4.0

ordered-set 4.1.0

orjson 3.10.7

oss2 2.17.0

packaging 24.1

pandas 1.5.3

parso 0.8.4

pexpect 4.9.0

pillow 10.4.0

pip 24.2

platformdirs 4.3.6

prettytable 3.11.0

prompt_toolkit 3.0.48

protobuf 4.25.5

psutil 6.0.0

ptyprocess 0.7.0

pure_eval 0.2.3

py-cpuinfo 9.0.0

pybind11 2.13.6

pycocotools 2.0.8

pycparser 2.22

pycryptodome 3.21.0

pydantic 2.9.2

pydantic_core 2.23.4

pydub 0.25.1

Pygments 2.18.0

pyparsing 3.1.4

python-dateutil 2.9.0.post0

python-multipart 0.0.12

pytorch-lightning 2.4.0

pytorch-wavelets 1.3.0

pytz 2023.4

PyWavelets 1.7.0

PyYAML 6.0.2

pyzmq 26.2.0

qudida 0.0.4

regex 2024.9.11

requests 2.28.2

resize-right 0.0.2

rich 13.4.2

ruff 0.6.9

safetensors 0.4.5

scikit-image 0.24.0

scikit-learn 1.5.2

scipy 1.14.1

seaborn 0.13.2

semantic-version 2.10.0

setuptools 60.2.0

shapely 2.0.6

shellingham 1.5.4

six 1.16.0

sniffio 1.3.1

sounddevice 0.5.0

stack-data 0.6.3

starlette 0.38.6

sympy 1.13.3

tabulate 0.9.0

tb-nightly 2.19.0a20241121

tensorboard 2.18.0

tensorboard-data-server 0.7.1

termcolor 2.5.0

terminaltables 3.1.10

thop 0.1.1-2209072238

threadpoolctl 3.5.0

tifffile 2024.9.20

timm 0.6.7

tokenizers 0.20.0

tomli 2.0.2

tomlkit 0.12.0

torch 2.1.2+cu121

torchaudio 2.1.2

torchmetrics 1.4.2

torchsummary 1.5.1

torchvision 0.16.2

tornado 6.4.1

tqdm 4.65.0

traitlets 5.14.3

transformers 4.45.2

triton 2.1.0

typer 0.12.5

typing_extensions 4.12.2

tzdata 2024.2

ultralytics 8.3.5

ultralytics-thop 2.0.9

urllib3 1.26.20

uvicorn 0.31.1

wcwidth 0.2.13

websockets 12.0

Werkzeug 3.0.4

wheel 0.44.0

xformers 0.0.23.post1

yapf 0.40.2

yarl 1.13.1

zipp 3.20.2
